# Localized Heat Therapy Improves Mitochondrial Respiratory Capacity but Not Fatty Acid Oxidation

**DOI:** 10.3390/ijms23158500

**Published:** 2022-07-31

**Authors:** Erik D. Marchant, Jamie P. Kaluhiokalani, Taysom E. Wallace, Mohadeseh Ahmadi, Abigail Dorff, Jessica J. Linde, Olivia K. Leach, Robert D. Hyldahl, Jayson R. Gifford, Chad R. Hancock

**Affiliations:** 1 Nutrition, Dietetics, and Food Science, Brigham Young University, Provo, UT 84602, USA; marchanterik@gmail.com; 2 Exercise Sciences, Brigham Young University, Provo, UT 84602, USA; nanikaluhiokalani@gmail.com (J.P.K.); taysomew@gmail.com (T.E.W.); ahmadi886@gmail.com (M.A.); adorff.16@gmail.com (A.D.); jessicajlinde@gmail.com (J.J.L.); olivia.k.leach@gmail.com (O.K.L.); robhyldahl@byu.edu (R.D.H.); jaysongifford@byu.edu (J.R.G.)

**Keywords:** skeletal muscle, exercise, mitochondria, heat stress, high-intensity interval training

## Abstract

AIM: Mild heat stress can improve mitochondrial respiratory capacity in skeletal muscle. However, long-term heat interventions are scarce, and the effects of heat therapy need to be understood in the context of the adaptations which follow the more complex combination of stimuli from exercise training. The purpose of this work was to compare the effects of 6 weeks of localized heat therapy on human skeletal muscle mitochondria to single-leg interval training. METHODS: Thirty-five subjects were assigned to receive sham therapy, short-wave diathermy heat therapy, or single-leg interval exercise training, localized to the quadriceps muscles of the right leg. All interventions took place 3 times per week. Muscle biopsies were performed at baseline, and after 3 and 6 weeks of intervention. Mitochondrial respiratory capacity was assessed on permeabilized muscle fibers via high-resolution respirometry. RESULTS: The primary finding of this work was that heat therapy and exercise training significantly improved mitochondrial respiratory capacity by 24.8 ± 6.2% and 27.9 ± 8.7%, respectively (*p* < 0.05). Fatty acid oxidation and citrate synthase activity were also increased following exercise training by 29.5 ± 6.8% and 19.0 ± 7.4%, respectively (*p* < 0.05). However, contrary to our hypothesis, heat therapy did not increase fatty acid oxidation or citrate synthase activity. CONCLUSION: Six weeks of muscle-localized heat therapy significantly improves mitochondrial respiratory capacity, comparable to exercise training. However, unlike exercise, heat does not improve fatty acid oxidation capacity.

## 1. Introduction

Mitochondria are central to the metabolic health of skeletal muscle. Increases in mitochondrial content and respiratory capacity are associated with improved endurance capacity, insulin sensitivity, and the preservation of muscle mass [1,2,3]. On the other hand, reductions in mitochondrial content and respiration are associated with conditions like insulin resistance, diabetes, and muscle atrophy [4,5,6,7,8,9]. Therefore, the mitochondria are a potential target to prevent or treat impaired muscle and overall health [10,11].

Exercise training is a potent stimulus for improved mitochondrial respiratory capacity and increased mitochondrial biogenesis [12,13]. Exercise training leads to hormetic adaptations in the mitochondria in response to a variety of types of stress including mechanical, hypoxic, energetic, oxidative, and heat [14]. For example, oxidative stress alone can activate peroxisome proliferator-activated receptor gamma coactivator 1-alpha (PGC-1α) in skeletal muscle, which is a primary regulator of mitochondrial biogenesis [15]. Energetic stress, which leads to the phosphorylation and activation of 5′ AMP-activated protein kinase (AMPK), has also been shown to activate pathways leading to increased oxidative capacity of the mitochondria [16,17]. While oxidative and energetic stress have received much attention regarding their effect on mitochondria, the effects of heat stress are not as well understood.

Heat stress alone has been shown to cause increases in mitochondrial content in cultured myotubes as well as rodent skeletal muscle [18,19]. In human subjects, repeated exposure to mild heat stress can signal for increases in mitochondria and improvements in overall muscle health. For example, acutely raising core and muscle temperature using a water-perfused suit activates signaling pathways associated with mitochondrial biogenesis and angiogenesis [20]. Furthermore, growing evidence suggests that mild heat stress can improve glucose management and other cardiometabolic parameters associated with insulin resistance and diabetes, some of which may be mediated by improved mitochondrial health [21,22,23].

Previous work from our research group has shown that mild heat stress can improve mitochondrial function and limit muscle atrophy that occurs during disuse in human subjects. Specifically, six consecutive days of heat exposure using short-wave diathermy, which raised internal muscle temperature to approximately 40 °C, was sufficient to increase mitochondrial respiratory capacity by about 30% [24]. During a period of 10 days of single-leg immobilization, our group also observed a 10.8% reduction in the cross-sectional area of individual myofibers from the vastus lateralis, accompanied by nearly a 30% reduction in mitochondrial respiratory capacity. Interestingly, when short-wave diathermy was applied for 2 h per day during this period of disuse, the change in cross-sectional area was limited to only a 5.8% reduction, with no change in mitochondrial respiration [25]. Furthermore, short-wave diathermy was sufficient to prevent reductions in vascular function and angiogenic markers [26].

The aforementioned work from our group suggests that short-wave diathermy, or more generally, mild heat stress, may have therapeutic benefits if applied in clinical situations like limb disuse or chronic metabolic disease. However, many clinical situations are not resolved in such a short time as 6 or 10 days. Therefore, studies of greater duration (weeks or months) are necessary to determine the utility of heat therapy over longer periods of time.

Few studies have directly compared the benefits of heat therapy to exercise training in human subjects, which is important to contextualize the benefits of heat stress. For example, fat oxidation capacity in muscle is greatly influenced by exercise training and is an important adaptation that improves endurance capacity, but whether heat stress improves fat oxidation is not known. Therefore, this study aimed to determine if 6 weeks of localized heat therapy (HT) increases mitochondrial respiratory capacity and content in human skeletal muscle, and to compare the effects of HT to those of single-leg interval training (EX). This study design was different from previous work from our group because heat was not applied on consecutive days over a short period of time (6 or 10 days), but rather 3 times per week over the course of 6 weeks. We hypothesized that HT would result in improved mitochondrial respiratory capacity and fat oxidation, which would be associated with an increase in mitochondrial respiratory protein content and enzyme activities. We also hypothesized that the benefits of HT would be less than those induced by the more complex combination of stimuli from EX.

## 2. Results

### 2.1. Baseline Characteristics

After excluding 6 subjects due to changes in schedule or failure to obtain muscle tissue from the baseline biopsy, 35 subjects (18 female, 17 male) were included in this study and randomly divided into 3 groups: Sham (*n* = 11), HT (*n* = 13), or EX (*n* = 11). No differences were observed between groups at baseline for maximal work rate during single-leg extension exercise (WRmax), age, height, weight, BMI, or skinfold thickness (Table 1).

### 2.2. Sham, HT, and EX Interventions

**Figure 1:**
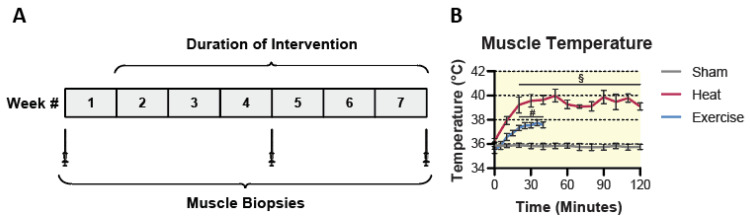
Study timeline (**A**), and internal muscle temperature during sham, HT and EX interventions (**B**). Statistical difference in the HT group compared to time 0 is indicated by § (*p* < 0.001). Statistical difference in the EX group compared to time 0 is indicated by # (*p* < 0.001). Results are represented as mean ± SEM (*n* = 7–8).

To assess the effects of either 6 weeks of HT or EX on muscle mitochondrial content and respiratory capacity, subjects visited the lab on 3 occasions each week for a total of 6 weeks (Figure 1A). On each visit, the HT group was subjected to a 2 h session of short-wave diathermy, localized to the right quadriceps muscles. The sham control group also received 2 h sessions of diathermy, but with the machine turned off. Subjects in the HT or sham groups were single-blinded to whether the diathermy unit was active or not. Previous results from our group suggest this is an effective sham because blinded subjects report an increase in the sensation of warmth regardless of whether the machine is active or not [25]. On each visit, the EX group performed 40 min of single-leg extension exercise on a modified cycle ergometer, with the intent of localizing the effects of exercise to the quadriceps muscles of the right leg. This exercise intervention involved four bouts of single-leg knee extension intervals interspersed with 4 min active recovery using a custom single-leg extension ergometer. Each bout of exercise was performed at 80% of WRmax, which was determined by a graded exercise test to exhaustion. We implemented this single-leg extension exercise protocol because it has been shown to be an extremely effective model for aerobic training, as it allows subjects to work at a higher muscle-specific VO_2_ than if they were performing a whole-body exercise [27]. Furthermore, another benefit of this model is that the effects of exercise are primarily isolated to the quadriceps muscles [28]. We observed a significant interaction between treatment and time (*p* < 0.0001) for internal muscle temperature of the vastus lateralis, which reached about 39.5 °C and 37.5 °C in the HT and EX groups, respectively. Post hoc analysis revealed that both the HT and EX groups experienced significant changes from baseline at the 20 min time point, and for the remainder of the treatment following that time point (*p* < 0.001). No change in muscle temperature was observed in the sham group (Figure 1B). We note that the goal of the HT and EX interventions was not to match the temperature change observed in the two groups. Rather, we sought to use a HT intervention that would generate at least as much heat as that which occurred with EX. Muscle biopsies were performed at baseline, 3 weeks, and 6 weeks, as shown in the study timeline in Figure 1A.

### 2.3. Work Rate Max

**Figure 2:**
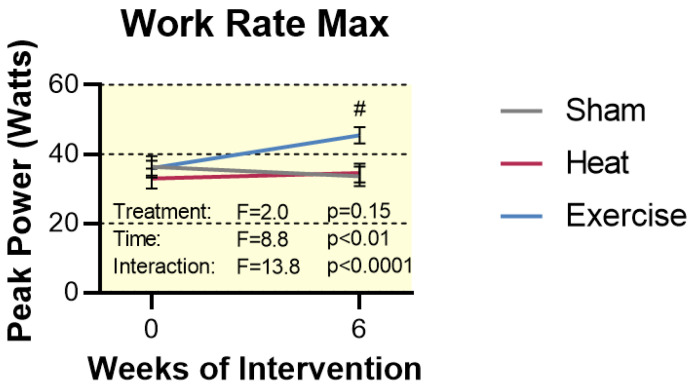
Work rate max attained during single-leg extension graded exercise test to exhaustion. Statistical difference in the EX group compared to time 0 is indicated by # (*p* <0.0001). Results are represented as mean ± SEM (*n* = 11–12).

Changes in exercise capacity were determined via a WRmax test performed on a modified cycle ergometer. This test was designed similarly to a typical VO_2_ max test; subjects began with a light warm-up, and then the work rate was increased 5–10 watts per minute until fatigue. Wrmax was determined at baseline, as well as after the full 6-week intervention. No changes were observed in the Sham or HT groups. However, EX caused a 27% increase from baseline, indicating this form of exercise was sufficient to improve skeletal muscle function (*p* < 0.0001, Figure 2).

### 2.4. Mitochondrial Content

**Figure 3:**
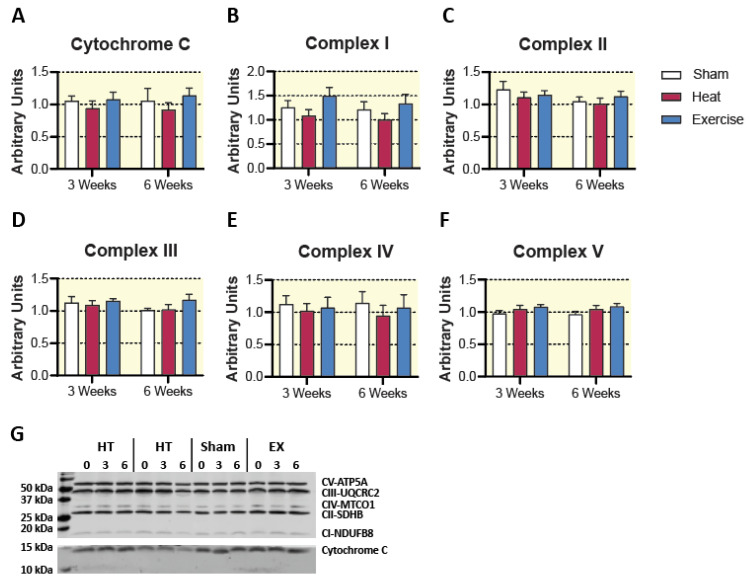
Mitochondrial protein content assessed by Western blot. (**A**–**F**) All individual values are reported as a fold change from their own baseline measure. Results are represented as mean ± SEM (*n* = 8–10). (**G**) Representative blots.

**Figure 4:**
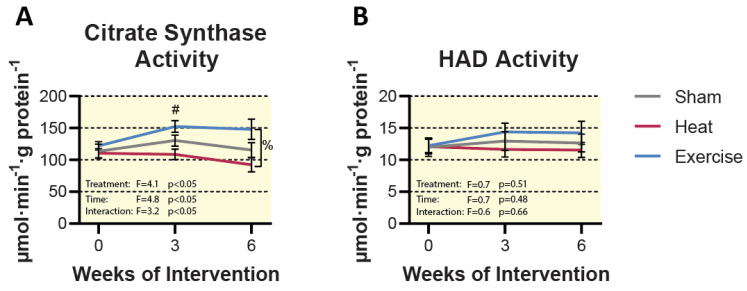
Citrate synthase and HAD activity. (**A**) Citrate synthase activity. (**B**) HAD activity. Statistical difference in the EX group compared to baseline is indicated by # (*p* < 0.05). Statistical differences between groups are indicated by % (*p* < 0.05). Results are represented as mean ± SEM (*n* = 8–10).

Neither HT nor EX increased markers of mitochondrial content (cytochrome c and mitochondrial complexes I–V) as assessed by Western blot (Figure 3). Enzyme activity of TCA cycle and beta-oxidation enzymes were assessed as markers of mitochondrial content. Hydroxyacyl-CoA dehydrogenase (HAD) activity was no difference between groups at any time point (Figure 4). Despite no change in mitochondrial respiratory proteins and HAD activity, we observed a significant treatment by time interaction for citrate synthase activity (*p* < 0.05). Post hoc analysis revealed a 25.1 ± 4.3% change from 0 to 6 weeks of EX treatment (*p* < 0.05), as well as a significant difference between HT and EX at 6 weeks (*p* < 0.05). Aside from these two individual differences, there were no significant pairwise differences between groups at any time point (Figure 4). The activity of citrate synthase, a key TCA cycle enzyme, is highly correlated with mitochondrial content [29]. Our results suggest that EX increased mitochondrial content, while HT did not. However, it should be acknowledged that without a direct measure of mitochondrial mass or volume, we cannot conclusively say that EX increased mitochondrial content [30]. Particularly, no changes were observed in mitochondrial respiratory protein content (Figure 3).

### 2.5. Mitochondrial Respiration

To better understand the effects of HT or EX on mitochondrial function, respiratory capacity of permeabilized muscle fibers was measured using high-resolution respirometry. The use of respirometry allows for a more complete assessment of mitochondrial respiratory function compared to individual enzyme activities, as it measures the capacity of intact mitochondria to consume oxygen under a variety of substrate/inhibitor conditions and relies on the coordinated function of a whole cascade of enzymes. We utilized two separate substrate/inhibitor titration protocols: the first to assess general mitochondrial respiratory capacity, and the second to assess fatty acid oxidation capacity by the mitochondria.

**Figure 5:**
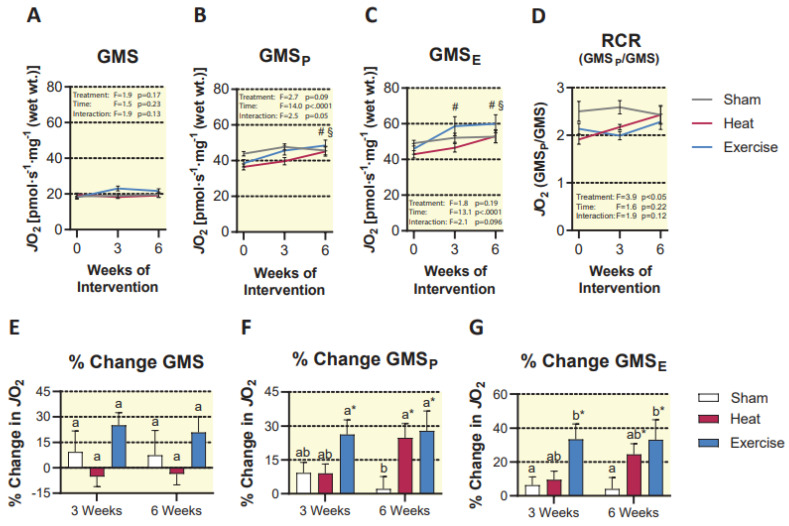
Mitochondrial respiration expressed as absolute respiration over the course of the intervention (**A–C**) and respiratory control ratio (**D**). Respiration expressed as a percent change from baseline in each group (**E**–**G**). Statistical difference in the HT group compared to baseline is indicated by § (*p* < 0.05). Statistical difference in the EX group compared to baseline is indicated by # (*p* < 0.05). In panels E-G, shared letters are no different from each other, and an asterisk (*) indicates a significant difference from baseline (*p* < 0.05). Results are represented as mean ± SEM (*n* = 8–11).

Our first titration protocol revealed a significant effect of time (*p* < 0.0001) and an interaction between the treatment group and time, which approached significance (*p* = 0.05) for maximal coupled respiration supported by glutamate, malate, succinate, and ADP (GMS_P_). Post hoc analysis revealed no differences between treatment groups at any time point, but a significant increase in both the HT and EX groups at 6 weeks compared to baseline (24.8 ± 6.2 % and 27.9 ± 8.7%, respectively; *p* < 0.05). Therefore, both HT and EX were effective at improving maximal coupled respiration (Figure 5). Consistent with this finding, uncoupled respiration (glutamate, malate, succinate, ADP, and FCCP) exhibited a significant effect of time (*p* < 0.0001) and an interaction between treatment group and time that approached significance (*p* = 0.093). Post hoc analysis revealed that EX caused a significant increase in uncoupled respiration at 3 weeks compared to baseline (33.4 ± 9.0%, *p* < 0.05). Furthermore, at 6 weeks, HT and EX were both significantly higher than their own baseline (24.5 ± 6.2% and 33.2 ± 11.7%, respectively; *p* < 0.05). Again, there were no significant pairwise differences between groups at any time point (Figure 5). We also observed that neither HT nor EX resulted in increased LEAK respiration (glutamate, malate, and succinate, without ADP) which is a measure of the amount of O_2_ consumption by the mitochondria which does not contribute to ATP production. This finding was supported by no change in respiratory control ratio (coupled/LEAK respiration) in either treatment group (Figure 5). This indirectly suggests that under conditions supported by glutamate, malate, and succinate, neither HT nor EX altered mitochondrial coupling of oxygen consumption to ATP production.

**Figure 6:**
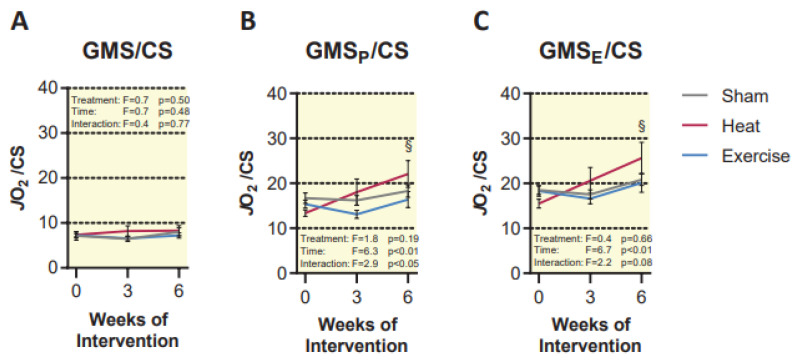
(**A**–**C**) Mitochondrial respiration normalized to citrate synthase activity. Units for this measure are nmol O_2_*s^−1^/U CS. Statistical difference in the HT group compared to baseline is indicated by § (*p* < 0.01). Results are represented as mean ± SEM (*n* = 8–10).

Due to the observation that HT caused an increase in mitochondrial respiratory capacity, but no change in CS activity, we suspect that the benefit of HT was due to an intrinsic improvement in respiratory capacity of existing mitochondria, and not an increase in total mitochondria. Thus, we normalized respiratory capacity to CS activity to determine if there was a difference in intrinsic respiration between groups (Figure 6). Interestingly, neither sham nor EX resulted in an intrinsic change in respiration, but there was an intrinsic change in coupled and uncoupled respiration in the HT group from pre- to post-intervention (*p* < 0.01, Figure 6).

**Figure 7:**
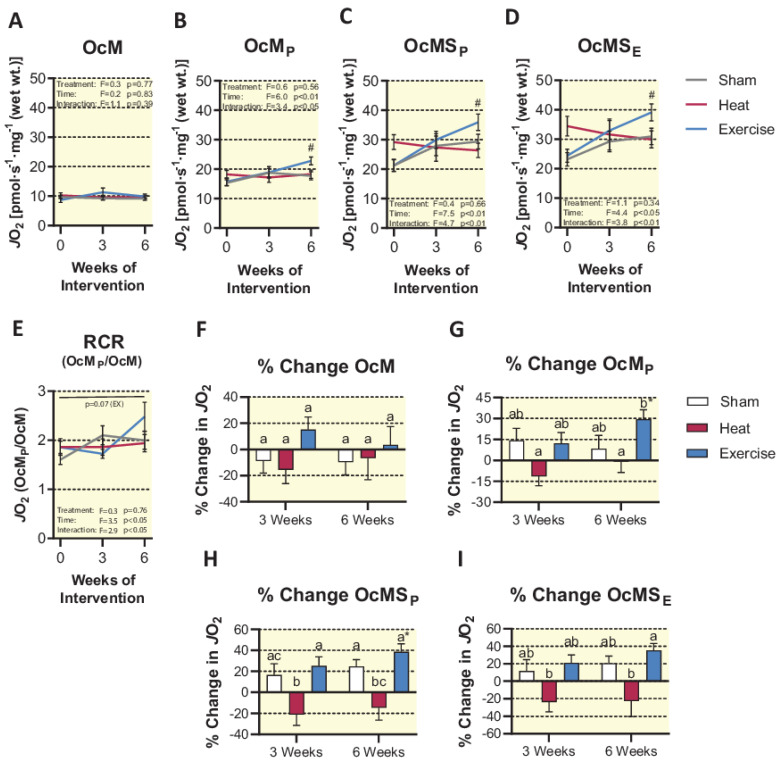
Mitochondrial fatty acid oxidation expressed as absolute respiration over the course of the intervention (**A–D**) and respiratory control ratio (**E**). Respiration expressed as a percent change from baseline in each group (**F–I**). Statistical difference in the EX group compared to baseline is indicated by # (*p* < 0.05). In panels (**E**–**G**), shared letters are no different from each other, and an asterisk (*) indicates a significant difference from baseline (*p* < 0.05). Results are represented as mean ± SEM (*n* = 9–10).

When mitochondrial respiratory capacity was measured under fatty acid supported conditions (octanoylcarnitine, malate, ADP), HT did not affect maximal coupled respiration. However, under this titration protocol, we observed a significant interaction between treatment and time (*p* < 0.05). Post hoc analysis revealed that EX caused a significant increase at 6 weeks compared to baseline (29.5 ± 6.8%, *p* < 0.01). This finding was consistent following the addition of succinate, where we observed a significant interaction of treatment and time (*p* < 0.01), as well as a 38.6 ± 7.7% increase in the EX group at 6 weeks compared to baseline (*p* < 0.001). Uncoupled respiration (octanoylcarnitine, malate, ADP, succinate, and FCCP) was also increased at 6 weeks compared to baseline in the EX group (35.4 ± 7.8%, *p* < 0.01), resulting in another significant interaction of treatment group and time (*p* < 0.01). Furthermore, at 6 weeks, EX trended toward an increase in the ratio of coupled fat oxidation to LEAK respiration (RCR), which approached significance (*p* = 0.07), along with a significant interaction of treatment group and time (*p* < 0.05), indicating an intrinsic improvement in coupling efficiency of the mitochondria when assessed under fatty acid-supported conditions (Figure 7). Altogether, these results suggest that EX improves fatty acid oxidation while HT does not.

## 3. Discussion

This study is the first to show the effects of localized heat therapy on mitochondrial respiratory capacity in human skeletal muscle compared to a single leg interval exercise training model. The main finding of this study was that HT and EX both increase mitochondrial respiratory capacity but only EX improves fat oxidation capacity. Furthermore, EX resulted in an increase in CS activity, as well as fat oxidation, but HT did not improve either measure. Given that CS activity is a common surrogate measure of mitochondrial content, and fat oxidation has been shown to be closely associated with mitochondrial content, this suggests that EX, but not HT increased mitochondrial content [29,31]. Under fatty acid-supported conditions, EX trended toward an improvement in RCR, which is the ratio between maximal coupled and LEAK respiration. As this ratio increases, it suggests there is an increase in substrate turnover, a decrease in proton leak, or a combination of the two, making it a useful tool to evaluate mitochondrial function or respiratory control [32]. Altogether, these results suggest that HT can improve mitochondrial respiratory capacity without necessarily increasing mitochondrial content. Furthermore, EX causes a significant improvement in mitochondrial respiratory capacity, which is associated with increased mitochondrial content and fatty acid oxidation. These findings are consistent with our hypothesis that HT improves mitochondrial respiration, but to a lesser degree than EX.

Previous work from our research group concluded that six consecutive days of HT using short-wave diathermy (2 h/day) results in nearly a 30% improvement in mitochondrial respiratory capacity compared to untreated muscle. This improvement in mitochondrial respiratory capacity was associated with increases in complexes I and V of the electron transport system, as well as a small increase in PGC-1α protein expression [24]. Furthermore, the ability of short-wave diathermy to improve mitochondrial health is clinically relevant because it mitigates some of the skeletal muscle atrophy that occurs during single-leg immobilization in human subjects. Specifically, 10 days of single-leg immobilization in young, active individuals resulted in a 10.8% decrease in cross-sectional area of skeletal muscle fibers and nearly a 30% reduction in mitochondrial respiratory capacity. However, in subjects receiving 2 h of short-wave diathermy treatment per day, muscle fiber size was only reduced by 5.8%, with no change in mitochondrial respiration [25]. It should be noted that future work should also determine if diathermy prevents a decline in skeletal muscle strength or fitness due to immobilization, as this was not a measure made in this previous study [25]. Though these rapid improvements in mitochondrial respiratory capacity from past studies were remarkable, one limitation of this previous work is that many clinical situations are not resolved in such a short time (i.e., long-term bed rest or athletic injuries). For this reason, the present study is valuable because it shows that a longer intervention (6 weeks), albeit less frequent (3 times/wk), also improves mitochondrial respiratory capacity in skeletal muscle.

Another reason why these results are important is that we have contextualized the benefits of HT by comparing directly to an exercise group. The use of this EX protocol was implemented because it is known to cause robust improvements in mitochondrial respiratory capacity and content [33,34]. Impressively, HT caused improvements in overall respiratory capacity which were no different from EX, suggesting that HT provides a potent stimulus for mitochondrial adaptation relative to EX. Of course, there were some differences between HT and EX, particularly in fat oxidation and citrate synthase activity, suggesting that only EX increased mitochondrial content, and not HT. This makes sense because exercise powerfully induces mitochondrial adaptations as a result of a complex combination of stimuli including calcium signaling, energetic, oxidative, hypoxic, and possibly thermic stress, which activate overlapping pathways [35]. For example, exercise results in the production of reactive oxygen species and other stress signals, which activate PGC-1α via p38 MAPK [35]. This pathway can be suppressed during exercise through supplementation with antioxidants, suggesting that some of the benefits of exercise are ROS-dependent [36,37,38]. Exercise also results in energetic stress due to elevated ATP turnover rates and a shift in the balance of high-energy phosphates and electron carriers (AMP:ATP and NAD^+^:NADH) [39]. Energetic stress during exercise leads to the activation of AMPK and the deacetylase SIRT1, both of which are known to regulate mitochondrial metabolism, and play a role in mitochondrial biogenesis [40,41,42]. This study, along with previous work suggests that thermic stress may be a contributor to mitochondrial adaptations during exercise training.

Thermic stress is a known activator of heat shock proteins (HSPs), which are suspected to play a role in mitochondrial health in skeletal muscle [42,43,44]. Specifically, work from our laboratory showed that HSP72 protein expression increases with short-wave diathermy treatment [24]. Furthermore, HSP72 is crucial for some metabolic improvements in muscle following heat therapy [44]. Overexpression of HSP72 also results in greater mitochondrial content in murine skeletal muscle [43]. Aside from the activation of HSP72, heat may improve mitochondrial respiratory capacity via various other pathways may contribute to the benefits of HT observed in this study. For example, short-wave diathermy has been shown to increase AMPK phosphorylation acutely, which may lead to mitochondrial improvements when repeated chronically [24]. Furthermore, heat stress can increase SIRT1 protein expression, which may augment PGC-1α activity and thus, mitochondrial content [18]. Heat is also likely to increase oxidative stress in skeletal muscle, which has been well studied in avian and porcine models [45,46,47,48]. Therefore, it is possible that short-wave diathermy leads to mild increases in oxidative stress, resulting in signaling for mitochondrial adaptation. Another potential mechanism that would be interesting to investigate is the role of the neuromuscular junction in heat-induced adaptations. Elevated muscle temperatures are known to impair neuromuscular transmission acutely [49]. However, when applied passively, heat stress may result in a preconditioning effect that protects the neuromuscular junction [50].

The observation that HT improves mitochondrial respiratory capacity is interesting, as it was not accompanied by a significant increase in mitochondrial content. While mitochondrial content and respiratory capacity often track together, this is not always the case [51]. Previous reports suggest that mitochondrial respiration may increase without changes in the amount of respiratory protein or mitochondrial volume [52]. Therefore, the use of high-resolution respirometry to assess mitochondrial respiratory capacity is valuable because it provides an integrative approach to evaluate a whole cascade of enzymes working in concert with one another. Some reasons why mitochondrial respiration can increase without changes in respiratory protein content or mitochondrial volume are changes in membrane lipid composition [53], the formation of mitochondrial super-complexes [54,55], or altered interactions between adjacent mitochondria [56]. While this study did not investigate the mechanistic reasons for improved respiratory capacity, this is an area for future research.

The greatest difference we observed between HT and EX was that EX alone was sufficient to improve fatty acid oxidation, but HT caused no improvement. Mechanistically, there are likely a variety of reasons why this difference occurred. As previously discussed, exercise is a potent stimulator of PGC-1α activity due to the convergence of several signaling pathways on this protein, leading to mitochondrial biogenesis [35]. PGC-1α is perhaps best known for its role as a coactivator for the peroxisome proliferator-activated receptor (PPAR) family of proteins which are nuclear receptor transcription factors that regulate the transcription of a variety of fatty acid transport and oxidation genes [57,58]. Furthermore, fatty acids are known ligands for PPARs, and are necessary for their transcriptional activity [59]. Exercise training, particularly endurance exercise is known to increase circulating free fatty acids in the blood [60,61]. Interestingly, even in situations outside of exercise, increasing fat availability for skeletal muscle is known to increase mitochondrial content and fat oxidation. For example, feeding mice a high-fat diet for 4–5 weeks has been shown to increase mitochondrial oxidative protein content, as well as mitochondrial respiratory capacity [62]. In humans, 56 days of lipid overfeeding was also sufficient to cause improved mitochondrial oxidative capacity in permeabilized muscle fibers, similar to the methods used in our present work [63]. Due to a likely increase in fat delivery to muscle during EX, it is unsurprising that fat oxidation capacity improved in our study. On the other hand, we have little reason to believe, given our results, that HT results in increased mobilization of fatty acids, or fat oxidation in the muscle. However, it should be noted that some heating modalities may result in a rise in free fatty acids in the blood, though certainly not the same degree as exercise [64,65].

While the benefits of HT on mitochondrial respiratory capacity observed in this study were perhaps not as robust as those of EX, we fully expect that HT has potential applications in a clinical setting. As previously mentioned, 10 days of short-wave diathermy can mitigate the skeletal muscle atrophy and mitochondrial impairment induced by single-leg immobilization in human subjects [25]. Furthermore, other methods of inducing heat stress, such as hot water immersion have been shown to improve insulin sensitivity and glucose management in both type 2 diabetic human subjects [22,66], as well as various animal models [21,67].

It should be addressed that not all studies involving passive heating of skeletal muscle result in beneficial mitochondrial adaptations. For example, several studies using different heating modalities do not report increases in mitochondrial content. However, it appears that whether whole-body heating or localized heating methods are employed, the key factor for eliciting changes in mitochondrial content is the degree to which internal muscle temperature rises during the intervention. Heating methods that cause muscle temperature to rise to or above 39–40 °C typically elicit mitochondrial adaptations [20,24,25], while methods not attaining this temperature typically do not [20,68,69]. Therefore, it is likely that there is a temperature threshold, which must be reached to induce changes in mitochondrial respiration or content.

Another area for future research would be to determine whether heat therapy has fiber type-specific effects. While this study did not aim to investigate fiber type-specific mitochondrial adaptations, it is plausible that there would be different effects on muscles containing differing ratios of type I vs. type II fibers. This study made measures only on tissue from the vastus lateralis, which is quite mixed in fiber type [70,71]. However, it should be noted that there is a wide range of variability between individuals resulting from differences in genetics, activity levels, age, or specific types of exercise training (endurance vs. resistance) [72,73,74]. Furthermore, type II fibers typically have fewer mitochondria, but have a high capacity to increase in mitochondrial content in response to exercise training [75,76]. Therefore, it is plausible that heat may also have a greater effect on type II fibers than on type I, though future work is needed to investigate this area.

Overall, this work shows that intermittent exposure to heat stress or single leg interval exercise training can improve skeletal muscle oxidative capacity. However, this method and duration of heating did not increase mitochondrial content, nor did it improve fat oxidation, which are typical adaptations that result from exercise training. Even so, building evidence from this work and others [22,25] suggests that heat therapy may be an effective intervention for improving muscle health. Future work should focus on the benefits of different heating modalities, lengths of treatment, and required temperatures to improve mitochondrial health in the context of metabolic disease and disuse.

## 4. Experimental Considerations

This work provides evidence that short wave diathermy can increase mitochondrial respiration when applied over the course of 6 weeks. We acknowledge that a limitation of this work is that the heat load between the HT and EX groups was not matched, and we did not include an exercising group that experienced no change in muscle temperature. Due to this limitation, we cannot determine if heat is a necessary part of the adaptations which occur due to exercise training. We also recognize that heat therapy may have some contraindications for certain clinical applications and suggest that future work should investigate the tolerability and feasibility of applying diathermy in such cases.

## 5. Materials and Methods

### 5.1. Subject Characteristics

Young, healthy volunteers (*n* = 41) were selected to participate in this study and were randomly assigned to receive a sham, heat, or exercise intervention 3 times per week over the course of 6 weeks. Both the heat and sham therapies were single-blinded. Subjects were sedentary, reporting an average of less than 3 h of formal physical activity per week. Six subjects were disqualified from the study because of a change in exercise habits, schedule or because no tissue was obtained from the baseline biopsy, resulting in a final number of 35 subjects. Subject characteristics are further detailed in Table 1. This study was approved by the Brigham Young University Institutional Review Board (Protocol F2020-023) and was conducted in accordance with the Declaration of Helsinki. Each subject was informed of the potential risks associated with the study, and written consent was obtained.

### 5.2. Study Description

Subjects were randomly assigned to a HT, EX, or sham group and provided a baseline muscle biopsy from the vastus lateralis of the right leg. Approximately one week following the initial biopsy, subjects began their assigned intervention, and reported to the lab 3 times per week for a total of 6 weeks. At both the 3- and 6-week time points, subjects provided another biopsy from the right leg, which took place at least 24–48 h after the previous exercise or heating session. Muscle biopsies were used for Western blotting, enzyme activity assays, and mitochondrial respiration assessments.

### 5.3. Exercise Protocol

Immediately following the baseline biopsy, participants were subjected to a single-leg extension exercise maximal exercise test to determine their knee-extension maximum work rate (WRmax). Participants were seated in an upright position with their right leg attached to a knee extension ergometer, which is a modified Monarch cycle ergometer. This method is previously described by Andersen et al. [28]. Participants were instructed to perform knee extensions at a rate of approximately 80 rpm and were given 3–5 min to warm up their leg. Following this initial warm-up, the resistance on the cycle ergometer was increased by 5–10 watts each minute until fatigue. WRmax was defined as the final power output which the subject was able to perform for a full minute. Following this initial test, subjects in the EX group reported to the lab 3 times per week for 40-min exercise sessions. Each training session consisted of a 6-min warm-up at 20% of WRmax, followed by four single-leg knee extension intervals (4 min at 80% of WRmax), with 4 min of recovery at 20% of WRmax between each interval, and a 6-min cool-down at 20% of WRmax. A WRmax test was also performed after the first 3 weeks of training to determine if an adjustment was needed for the difficulty of each workout.

### 5.4. Localized Heat Therapy

Localized heating to the quadriceps muscles of the right leg was performed using pulsed shortwave diathermy (Megapulse II; Accelerated Care Plus; Reno, NV, USA) for 2 h/day, 3 days/week. The diathermy units were set at 27.12 MHz and 800 pulses per second, with a pulse duration of 400 μs. Two Megapulse II units were used to heat the quadriceps muscles of each subject, and the units were slightly moved every 20 min to better heat the entire quadriceps muscle group. Muscle temperature during heating was expected to rise to approximately 40 °C, which has been previously shown in our laboratory [24].

### 5.5. Sham Therapy

Sham heating sessions were used for the control group, with the barrels of the diathermy units place on the quadriceps muscles, but with the machines turned off. As reported by Hafen et al., this method has been shown to cause an increase in the sensation of warmth on the skin, but does not cause an increase in muscle temperature [24].

### 5.6. Muscle Temperature

During the first day of the randomly assigned intervention, internal muscle temperature of the vastus lateralis was measured for either 40 min of EX, or 2 h of HT or sham therapy. First, the skin over the vastus lateralis was cleaned with chlorhexidine and a local anesthetic (1% lidocaine with epinephrine) was used to numb the area. An 18-gauge, 1.88 in. Cathlon I.V. catheter (Smiths Medical; Minneapolis, MN, USA) was inserted approximately 3.5 cm into the vastus lateralis. A small thermocouple (IT-17:3; Physitemp Instruments; Clifton, NJ, USA) was passed through the catheter, and into the muscle. Once the thermocouple was inserted, the catheter was removed, and temperature was recorded using an Iso-Thermex machine (#256 Columbus Instruments; Columbus, OH, USA). In order to prevent injury from the thermocouple heating up during diathermy, the thermocouple was wrapped in gauze during the heat intervention.

### 5.7. Muscle Biopsies

Percutaneous muscle biopsies were performed at 3 time points during the study: baseline, 3 weeks, and 6 weeks. Biopsies were taken from the vastus lateralis of the right leg using the Bergström technique, which is thoroughly described by Shanely et al. [77]. Briefly, the skin was sterilized using chlorhexidine, and the site was anesthetized using 1% lidocaine with epinephrine. An incision (~1 cm) was made in the skin and into the muscle, and a Bergström biopsy needle was inserted about 3 cm deep into the muscle. A small piece of muscle (~75–150 mg) was removed using manual suction, and about 20 mg of tissue was place in ice-cold BIOPS buffer (60 mM K-MES, 35 mM KCl, 7.23 mM K_2_EGTA, 2.77 mM CaK_2_EGTA, 20 mM imidazole, 0.5 mM DTT, 20 mM taurine, 5.7 mM ATP, 15 mM PCr, and 6.56 mM MgCl_2_) for respiratory analysis. The remaining tissue was snap-frozen liquid nitrogen for protein analysis and activity assays.

### 5.8. Mitochondrial Respiration

Small bundles of skeletal muscle fibers (2–5 mg) were blotted, weighed, and gently teased apart using fine-tipped forceps to partially separate fibers without removing them from the fiber bundle. Each fiber bundle was rotated for 30 min at 4 °C in BIOPS buffer containing 50 μg/mL saponin to selectively permeabilize cell membranes. Fiber bundles were then rinsed for 15 min in ice-cold MiR05 buffer (110 mM sucrose, 60 mM potassium lactobionate, 2 mM MgCl_2_, 20 mM taurine, 10 mM KH_2_PO_4_, 0.5 mM EGTA, 20 mM HEPES, and 1 g/l bovine serum albumin). Following the rinse step, bundles were placed inside the Oroboros Oxygraph-2k (O2k) with MiR05 buffer, set at 37 °C with the stir bars spinning at 750 revolutions/min. Supplemental oxygen was added to each chamber to maintain O_2_ concentrations between 500 and 200 μM throughout the experiment. Substrates were added using the following 2 titration protocols to measure mitochondrial respiration:

*Titration Protocol 1:* Glutamate (10 mM final concentration), malate (2 mM) and succinate (10 mM) were added to stimulate LEAK respiration (GMS), which is described by Pesta and Gnaiger [78]. Following this, ADP was titrated in steps (12.5–10,000 µM) to determine respiratory kinetics and maximal coupled respiration (GMS_P_). Cytochrome c (10 µM) was used as a quality control to ensure that the outer mitochondrial membrane was intact. An increase in oxygen consumption >10% following the addition of cytochrome c was used as an indication that a given sample should be excluded from analysis. FCCP was then titrated (0.5 µM steps) to uncouple respiration (GMS_E_). Antimycin A (2.5 µM) was added to inhibit CIII and determine background oxygen consumption. Any remaining signal was subtracted from the previous measures. Due to the variability of weighing and teasing apart muscle fibers, measures were made in duplicate, where possible, to ensure accurate results.

*Titration Protocol 2:* Octanoylcarnitine (0.5 mM) and malate (2 mM) were used to stimulate LEAK respiration (OcM), followed by ADP (2.5 mM) to induce maximal fatty-acid oxidation (OcM_P_). Succinate (10 mM) was then added (OcMS_P_), followed by cytochrome c (10 µM) to ensure mitochondrial membrane integrity. FCCP was added next to stimulate uncoupled fatty-acid oxidation (OcMS_E_), and antimycin A (2.5 µM) to determine background oxygen consumption.

### 5.9. Tissue Homogenization

Frozen tissue samples were weighed and homogenized using a glass-on-glass homogenizer in ice-cold T-PER Tissue Protein Extraction Reagent (78510; Thermo Fisher Scientific; Waltham, MA, USA) at a ratio of 9 µL buffer to 1 mg tissue. The homogenization buffer was supplemented with Halt Protease and Phosphatase Inhibitor Cocktail (1861280; Thermo Fisher Scientific; Waltham, MA, USA) per the manufacturer’s instructions. The homogenate was centrifuged at 1000 rcf at 4 °C for 10 min, and the protein concentration was determined using bicinchoninic acid protein assay methods (23225, Thermo Fisher Scientific; Waltham, MA, USA). The supernatant was stored at −80 °C until further analysis.

### 5.10. Citrate Synthase and HAD Activity

For citrate synthase activity, whole muscle homogenate (2 µL) was added in triplicate to a 96-well plate with 180 µL of 200 mM Tris buffer (0.2% Brij-37), 4 µL of 15 mM acetyl-CoA and 4 µL of 5 mM DTNB. Absorbance was measured at 412 nM once every 30 s for 5 min. Next, 10 mM oxaloacetate (10 µL) was added, and the plate was read at 412 nM once every 30 s for 5 min. Data points were fit in a linear fashion for both readings, and slopes were determined. The slope prior to the addition of oxaloacetate was subtracted from the slope that was measured after the addition of oxaloacetate. For HAD activity, the same procedure was followed, but NADH was added to a final concentration of 250 µM, and the plate was read at 340 nM once every 30 s for 5 min. Following this initial background measure, acetoacetyl-CoA was added to a final concentration of 100 µM, and the plate was read at the same intervals. Data points were fit in a linear fashion for both readings, and slopes were determined. The slope prior to the addition of acetoacetyl-CoA was subtracted from the slope that was measured after the addition of acetoacetyl-CoA. Bicinchoninic acid methods were used to correct all readings for total protein.

### 5.11. Western Blotting

Western blot samples were made up from muscle homogenate supernatant at equal protein concentrations in Laemmli buffer and heated at 45 °C for 5 min. Proteins were run on sodium dodecyl sulfate–polyacrylamide gels (4% stacking and 15% resolving) for 30 min at 100 V followed by 60 min at 150 V. Thirty micrograms of protein were loaded in each well of the gel. Following electrophoresis, separated proteins were transferred to nitrocellulose membranes (1620112; Bio-Rad; Hercules, CA, USA) at 100 V for 60 min in ice-cold transfer buffer. Following the protein transfer, the membranes were stained for total protein to ensure equal loading using Ponceau S BioReagent (P3504, Sigma-Aldrich; St. Louis, MO, USA). Each membrane was blocked for 60 min in 5% bovine serum albumin in tris-buffered saline, 0.1% TWEEN 20 (TBST), followed by an overnight application of total OXPHOS primary antibody cocktail (1:1000; ab110413; Abcam) or cytochrome c antibody (1:500; C5723; Sigma-Aldrich) diluted in 1% bovine serum albumin in TBST at 4 °C. Membranes were washed in TBST, and membranes were incubated for 60 min at RT in anti-mouse IgG secondary antibody (1:10,000; IRDye 926-65010; LI-COR Biosciences; Lincoln, NE, USA), diluted in 1% bovine serum albumin in TBST. Membranes were again washed in TBST and imaged using direct infrared imaging (Odyssey CLx, LI-COR Biosciences; Lincoln, NE, USA). All protein values were normalized within subject to their own baseline measure and expressed as a fold difference from baseline.

### 5.12. Statistics

All results were analyzed using a mixed ANOVA (3 different groups and 3 within-subject time point measures). Tukey HSD post hoc analysis was used to determine pairwise differences. Normality of each data set was determined using a Shapiro–Wilk test. When necessary, data were log-transformed, and confirmed for normal distribution prior to ANOVA analysis. All significant differences were tested with an alpha level of 0.05, which was set a priori.

## Figures and Tables

**Table 1:** Subject Characteristics.

	Age (y)	Height (cm)	Weight (kg)	BMI	Skinfold Thickness (mm)	Baseline WR Max (watts)
Sham (M = 6, F = 5)	20.5 ± 0.5	172.3 ± 3.5	61.5 ± 3.4	20.6 ± 0.7	21.0 ± 3.2	35.0 ± 2.5
HT (M = 5, F = 7)	21.8 ± 1.3	168.3 ± 1.8	62.3 ± 3.0	22.0 ± 1.0	22.2 ± 2.2	34.2 ± 2.4
EX (M = 4, F = 4)	22.7 ± 1.7	170.3 ± 2.5	67.7 ± 4.8	23.1 ± 1.3	26.3 ± 3.2	36.8 ± 2.2

All values are expressed as mean ± SEM.

## Data Availability

The data that support these findings are available upon request from the corresponding author.
